# Optical Coherence Tomography Imaging and Angiography of Skull Base Tumors Presenting as a Middle Ear Mass in Clinic

**DOI:** 10.3390/diagnostics15060732

**Published:** 2025-03-14

**Authors:** Dorothy W. Pan, Marcela A. Morán, Wihan Kim, Zihan Yang, Brian E. Applegate, John S. Oghalai

**Affiliations:** 1 Caruso Department of Otolaryngology—Head and Neck Surgery, University of Southern California, Los Angeles, CA 90033, USA; 2 Alfred Mann Department of Biomedical Engineering, University of Southern California, Los Angeles, CA 90089, USA

**Keywords:** optical coherence tomography, angiography, skull base tumors, middle ear masses, glomus jugulare, cholesteatoma, facial nerve schwannoma

## Abstract

**Background**: Skull base tumors can extend into the temporal bone and occasionally even be visible through the tympanic membrane (TM) if they grow into the middle ear cavity. The differential diagnosis of a skull base mass is extensive and ranges from non-tumorous lesions like cholesteatoma to benign tumors like schwannoma and to malignant lesions like metastatic cancer. Optical coherence tomography (OCT) is a noninvasive imaging technique that can image tissue with high resolution in three dimensions, including through structures such as the TM and bone. OCT angiography is also able to assess tissue vascularity. We hypothesized that OCT could help shrink the differential diagnosis in clinic on the day of initial presentation. Specifically, we thought that OCT angiography could help distinguish between highly vascular skull base tumors such as glomus jugulare and other less vascular tumors and middle ear pathologies such as cholesteatoma and schwannoma. **Objectives**: We sought to determine whether OCT can image through the TM in clinic to distinguish a normal ear from an ear with a mass behind the tympanic membrane. Furthermore, we sought to assess whether OCT angiography can detect vascularity in these masses to help inform the diagnosis. **Methods**: We designed and built a custom handheld OCT system that can be used like an otoscope in clinic. It is based off a 200 kHz swept-source laser with a center wavelength of 1310 nm and a bandwidth of 39 nm. It provides a 33.4 μm axial and 38 μm lateral resolution. Cross-sectional images of the middle ear space, including OCT angiography, were captured in an academic neurotology clinic. Patients with normal ear exams, glomus tumors, cholesteatomas, and facial nerve schwannoma were imaged. **Results**: OCT images revealed key structures within the middle ear space, including the TM, ossicles (malleus and incudostapedial joint), chorda tympani, and cochlear promontory. OCT also identified middle ear pathology (using pixel intensity ratio in the middle ear normalized to the TM) when compared with patients with normal ear exams (mean 0.082, *n* = 6), in all patients with a glomus tumor (mean 0.620, *n* = 6, *p* < 0.001), cholesteatoma (mean 0.153, *n* = 4, *p* < 0.01), and facial nerve schwannoma (0.573, *n* = 1). OCT angiography revealed significant vascularity within glomus tumors (mean 1.881, *n* = 3), but minimal vascularity was found in normal ears (mean 0.615, *n* = 3, *p* < 0.05) and ears with cholesteatoma (mean 0.709, *n* = 3, *p* < 0.01), as expected. **Conclusions**: OCT is able to image through the TM and detect middle ear masses. OCT angiography correctly assesses the vascularity within these masses. Thus, OCT permits the clinician to have additional point-of-care data that can help make the correct diagnosis.

## 1. Introduction

Middle ear masses, including skull base tumors that extend into the middle ear, often present with ear complaints such as hearing loss and tinnitus, prompting ear examination. Usually, these tumors will have subtle findings seen on clinical ear exams that prompt additional imaging such as computed tomography (CT) and/or magnetic resonance imaging (MRI) scans. The patient then returns to the clinic to review the scans, and hopefully, a diagnosis can then be made. This process requires multiple visits over several weeks. A point-of-care diagnostic technique would thus be of tremendous benefit, both in reducing patient anxiety and potentially in reducing the need to order one or more imaging studies when there is no pathology found in the middle ear. Optical coherence tomography (OCT) is a noninvasive optical imaging technique that uses backscattered light from a laser source to rapidly scan tissues, thus acquiring a volumetric three-dimensional (3D) image. OCT can provide three-dimensional volumetric measurements, which could provide additional data to serve as an adjunct for ear examination. OCT has the potential to image middle ear structures through the TM with penetration to the cochlear promontory (CP) [1,2]. OCT has been used mainly experimentally as a diagnostic tool for otologic disease but has been well established for use clinically in ophthalmology for corneal, lens, and retinal imaging. Given the success of OCT for ophthalmological diagnoses, we sought to test the feasibility of using OCT to enable point-of-care diagnostics in the clinic exam room to distinguish between different types of middle ear masses.

In addition to 3D spatial imaging, OCT can assess for temporal changes in vivo by calculating the speckle variance in sequential scans to provide angiography data [3]. This dynamic imaging method can detect blood flow in tissues and can clearly demonstrate microvasculature. OCT angiography has been demonstrated to accurately map vasculature in intradermal tumor xenografts in mice [4]. Furthermore, in humans with subungual glomus tumors, OCT has been able to demonstrate tumor vascularity [5]. Therefore, OCT angiography is well positioned to detect vascular tumors such as glomus jugulare tumors behind the TM and to distinguish them from other less vascular types of middle ear masses such as cholesteatomas.

OCT has several potential advantages over traditional clinical examination techniques such as otomicroscopy, including (1) its ability to collect a 3D image rather than a 2D picture, (2) its ability to measure and quantify structures and masses, and (3) its potential ability to measure tumor vascularity with OCT angiography. In our study, we use both volumetric OCT and OCT angiography imaging through the TM in the clinic setting to image patients with normal ears and middle ear masses including glomus tumors, cholesteatomas, and facial nerve schwannoma. We hypothesize that the analysis of these volumetric and angiography images can provide additional information regarding (1) the status of a patient’s middle ear (is there a mass?) and (2) the potential identity of the mass (is it vascular or avascular?) beyond otomicroscopy alone.

## 2. Materials and Methods

A custom handheld OCT (HHOCT) device was built as recently described and used for the imaging performed for this study [6], which is similar to our previously published otoscopic OCT system [7]. Briefly, it contains a swept-source laser operating at 1310 nm and 39 nm bandwidth with a 200 kHz sweep rate providing a 33.4 μm axial and 38 μm lateral resolution in tissue. The z dimension pixel spacing was corrected assuming an index of refraction in air of 1.0. Measurements were performed and corrected using the refractive index in air of 1.0 and assuming a refractive index in tissue of 1.4 [8]. Therefore, in the z dimension, a 1 mm optical pathlength that is measured for air is 1 mm, while that for tissue is 1/1.4 mm. The handheld imaging probe contains an attachment for a disposable otoscope tip to fit on, so that it can be used just like a regular otoscope in the ear canal. An integrated otoscopic video is used to visualize the tympanic membrane, which is viewed on an endoscopy cart. A wireless foot pedal initiates data acquisition which collects otoendoscopy images as well as the OCT volume using our custom software written in Python 3.9.7.

Adult patients 18 years and older were recruited in our academic tertiary care neurotology outpatient clinic setting. For this project, images were collected of 20 ears from patients with diagnoses of glomus tumor, cholesteatoma, facial nerve schwannoma with a middle ear component, and normal ear exams as detailed in Supplementary Table S1. The nature of the noninvasive examination was discussed with our patients, and informed consent was obtained prior to research imaging. This study was approved by the University of Southern California Institutional Review Board (approval number HS-17-01014).

Capture of a volumetric scan is completed in less than 1 s, and several volume scans can be performed within a few seconds. Cross-sectional images of the middle ear space can be generated from the volumetric data with Matlab (MathWorks, Natick, MA, USA) and image analysis software (Amira, Thermo Fisher Scientific, Waltham, MA, USA) to visualize beyond the tympanic membrane down to the cochlear promontory, including ossicular structures, in patients with normal ear exams. The XY enface images showing the topography of the tympanic membrane with the malleus were generated as an averaged intensity projection over 40–50 pixels. In patients with abnormal ear exams such as cholesteatomas or tumors, the cross-sectional images can provide additional details about the status of the middle ear.

Representative cross-sectional images of each scan were taken with reference to the malleus umbo as a landmark. The mean pixel intensity in the region of interest within the middle ear mass in the mesotympanum below the tympanic membrane was quantified and normalized to the tympanic membrane mean pixel intensity in Fiji (ImageJ 1.54j, NIH open-source software, Bethesda, MD, USA). The normalization of pixel intensity to a known structure in the same image is necessary due to the possibility of variation in pixel intensity from different volume images due to variations in focal plane or scattering between patients. In normal ears, the region between the tympanic membrane and cochlear promontory at a similar depth as the middle ear masses below the tympanic membrane was quantified and normalized to the tympanic membrane mean pixel intensity.

For visualizing blood vessels, OCT angiography measurements were obtained by employing a set number of consecutive B-scans to assess the speckle variance at the identical location within the OCT morphology intensity. The total image size for the volumetric OCT angiography was 417 × 1280 × 2048 pixels and was captured approximately within 4.4 s. The rapid angiography scan allowed minimization of motion artifacts, which were not corrected for in the image analysis performed. The speckle variance in the OCT images was calculated as previously described [9] and analyzed in MatLab (MathWorks, Natick, MA, USA) to generate the OCT angiography images. Analogous to the volumetric data, the OCT angiography signal was quantified using Fiji by determining the mean pixel intensity in a 100 × 100 pixel box in the region of interest of the middle ear mass, normalized to the mean pixel intensity in a 50 × 50 pixel box over the lateral process of the malleus. In normal ears, a similar region was quantified in the mesotympanum. In ears with cholesteatoma and facial nerve schwannoma, the area of the tumor in the ear was identified with a combination of otoscopic image and volumetric OCT. For cholesteatomas, the area of the angiography signal was in the posterior mesotympanum or epitympanum. For the facial nerve schwannoma, the area of the angiography signal was in the posterior mesotympanum.

Statistical analysis was performed in Excel (Microsoft) using an unpaired Student’s *t*-test with statistical significance set to *p* < 0.05.

## 3. Results

OCT imaging was performed on a total of 20 ears from adult patients 18 years and older in our academic tertiary care neurotology outpatient clinic setting. These subjects included normal ears and pathologic ears of patients with diagnoses of glomus tumor, cholesteatoma, and facial nerve schwannoma with a middle ear component, as detailed in Supplementary Table S1. High-resolution volumetric OCT scans of the middle ear space were captured, processed, and analyzed to provide both 3D and cross-sectional OCT images. Structures including the tympanic membrane, ossicles, chorda tympani, and cochlear promontory were identified. Any middle ear masses were also identified. OCT data acquisition is rapid, and analysis of the volumes and cross-sectional images by an experienced clinician or researcher can be conducted within a couple of minutes. However, the OCT angiography images take additional time to analyze and post-process to generate the angiography images. These were carried out offline later in the day.

### 3.1. Volumetric OCT Representative Examples

#### 3.1.1. Normal Ear

**Figure 1:**
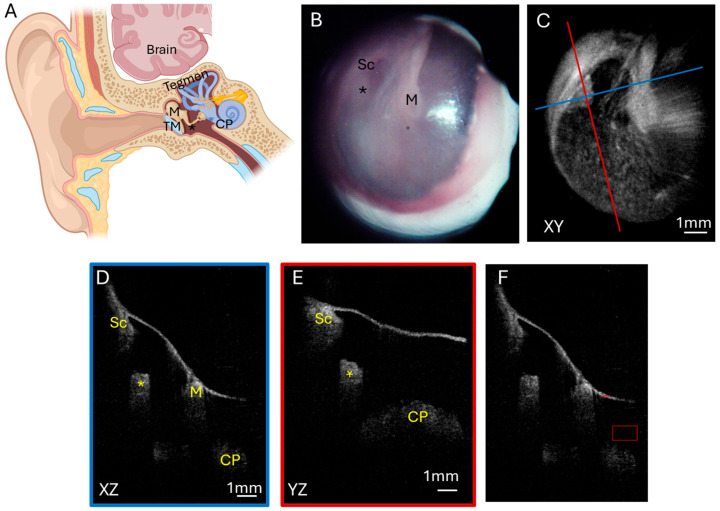
Normal ear. (**A**) Illustration of a normal ear showing the tympanic membrane, ossicular chain, and inner ear. The tegmen is bone that separates the ear from the brain temporal lobe sitting above the ear. Endoscopic view of the right tympanic membrane (**B**) of a patient with a normal ear exam. OCT enface XY view (**C**) of the tympanic membrane, with the blue line representing the plane leading to the middle ear for the cross-sectional XZ (**D**) view (blue box) and the red line representing the plane leading to the middle ear for the cross-sectional YZ (**E**) view (red box) showing the middle ear ossicles and cochlear promontory. (**F**) Quantification of pixel intensity in the mesotympanum (larger red box) in comparison to tympanic membrane pixel intensity (smaller red box). TM, tympanic membrane; M, malleus; Sc, scutum; *, incudostapedial joint; CP, cochlear promontory.

A normal ear (Figure 1A) has a translucent tympanic membrane (TM) with an aerated middle ear that contains the ossicular chain, containing the malleus, incus, and stapes. The temporal lobe of the brain sits over the ear with tegmen, a thin layer of bone, separating the ear and the brain. A total of six normal ears were analyzed. A representative endoscopic image of a patient with a normal ear (Figure 1B) demonstrates a translucent TM such that the incudostapedial joint can be visualized through the TM. The corresponding OCT enface XY (Figure 1C) view at the level of the TM is shown, with the blue line representing the plane going into the middle ear for the XZ (Figure 1D) cross-sectional view and the red line representing the plane going into the middle ear for the YZ (Figure 1E) cross-sectional view. A demonstration of the region where pixel intensity was quantified and normalized to tympanic membrane pixel intensity (Figure 1F) is also shown. The cross-sectional images demonstrate the location of the TM and an aerated middle ear space with signal penetration down to the cochlear promontory. Additionally, structures including the scutum bone underlying the posterior–superior edge of the TM and the ossicles, including the malleus and incudostapedial joint, are well visualized.

#### 3.1.2. Glomus Tumor

**Figure 2:**
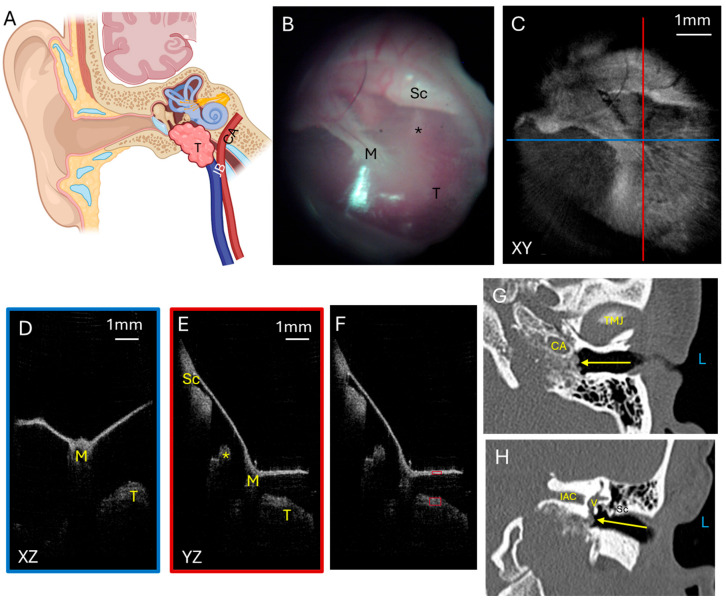
Glomus tumor. (**A**) Illustration showing a glomus jugulare tumor growing superiorly from the jugular bulb into the hypotympanum and mesotympanum. Not shown is that the tumor can grow posteriorly to erode skull base bone and invade into the posterior fossa to compress the cerebellum and brainstem. Endoscopic view of the left tympanic membrane (**B**) of a patient with a glomus jugulare with a small component of the tumor in the hypotympanum and mesotympanum. OCT enface XY view (**C**) of the tympanic membrane, with the blue line representing the plane leading to the middle ear for the cross-sectional XZ (**D**) view (blue box) and the red line representing the plane leading to the middle ear for the cross-sectional YZ (**E**) view (red box) showing the middle ear ossicles and tumor. (**F**) Quantification of tumor pixel intensity (larger red box) in comparison to tympanic membrane pixel intensity (smaller red box). Axial CT (**G**) showing the tumor (arrow) in the mesotympanic space, and coronal CT (**H**) showing the tumor (arrow) in the mesotympanic space invading the inner ear. L, left side; M, malleus; Sc, scutum; *, incudostapedial joint; T, tumor; TMJ, temporomandibular joint; CA, carotid artery; V, vestibule; IAC, internal auditory canal; JB, jugular bulb.

Glomus tumors are highly vascular benign skull base tumors. Glomus jugulare tumors arise from glomus cells within the jugular bulb. Often, these tumors grow superiorly from the jugular bulb into the middle ear such that a portion of the tumor can be visualized as a mass with a reddish hue in the middle ear behind the TM (Figure 2A). Glomus tympanicum tumors are smaller and arise from glomus cells within the middle ear. Both types tend to be slow growing but locally destructive, eroding through bone in the middle ear and skull base to extend intracranially to compress the temporal lobe if it grows superiorly or the cerebellum and brainstem if it grows posteriorly. Five patients had glomus jugulare tumors, two of which had previously been treated with radiation. One patient had a glomus tympanicum. Figure 2 shows a representative patient with a glomus jugulare tumor that had intracranial extension and a small hypotympanic and mesotympanic component visible in the middle ear space. The endoscopic image (Figure 2B) with the corresponding OCT enface XY (Figure 2C) view at the level of the TM are shown. The blue line corresponds with the plane going into the middle ear for the XZ (Figure 2D) cross-sectional view, and the red line corresponds with the plane going into the middle ear for the YZ (Figure 2E) cross-sectional view. These cross-sectional views clearly demonstrate the tumor present in the middle ear space beneath the TM. The tumor limited penetration to the cochlear promontory, but the ossicular chain is still visualized. The area of tumor in the middle ear space is the region where pixel intensity was quantified, normalized to tympanic membrane pixel intensity (Figure 2F). The axial CT (Figure 2G) and coronal CT (Figure 2H) demonstrate the bony destruction caused by the glomus jugulare tumor, with the slices showing the tumor in the mesotympanum with invasion into the otic capsule (Figure 2H, arrow).

**Figure 3:**
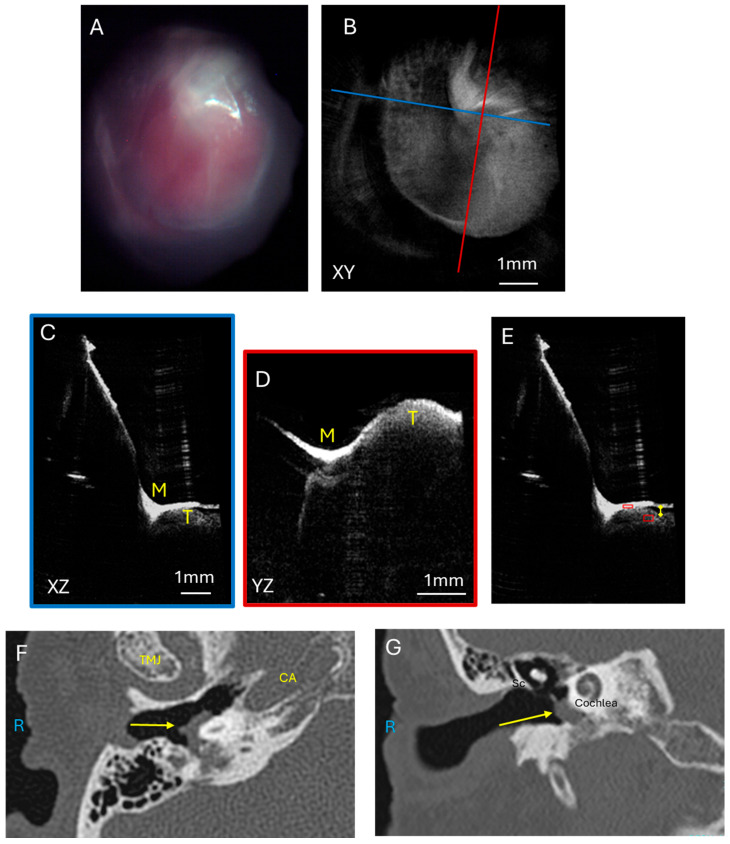
Glomus tumor. Endoscopic view of the right tympanic membrane (**A**) of a patient with a glomus jugulare with a large component of the tumor in the hypotympanum and mesotympanum. OCT enface XY view (**B**) of the tympanic membrane, with the blue line representing the plane leading to the middle ear for the cross-sectional XZ (**C**) view (blue box) and the red line representing the plane leading to the middle ear for the cross-sectional YZ (**D**) view (red box) showing the tumor which is touching the tympanic membrane and obstructing visualization of the middle ear ossicles. (**E**) Quantification of tumor pixel intensity in comparison to tympanic membrane pixel intensity (red boxes), and measurement of largest distance from the tumor to the TM (yellow line). Axial CT (**F**) showing the tumor (arrow) in the mesotympanic space, and coronal CT (**G**) showing the tumor (arrow) in the mesotympanic space. R, right side; M, malleus; Sc, scutum; T, tumor; TMJ, temporomandibular joint; CA, carotid artery.

Another patient had a more extensive glomus jugulare tumor in the middle ear and ear canal. This tumor signal was visualized just below the tympanic membrane. This is demonstrated in Figure 3 with the endoscopic image (Figure 3A) showing the red hue of the vascular tumor behind the tympanic membrane and the corresponding OCT enface XY (Figure 3B) view at the level of the TM. The blue line corresponds with the plane going into the middle ear for the XZ (Figure 3C) cross-sectional view, and the red line corresponds with the plane going into the middle ear for the YZ (Figure 3D) cross-sectional view. These cross-sectional views show the tumor immediately underneath the TM and even pushing on the TM (most evident in Figure 3D). Due to the tumor mass, the cochlear promontory and incudostapedial joint are obscured from view on OCT imaging. Quantification of pixel intensity in the area of the tumor in the middle ear space normalized to tympanic membrane pixel intensity was performed and is shown in Figure 3E. The axial CT (Figure 3F) and coronal CT (Figure 3G) show a larger component of the tumor in the middle ear space in this patient, concordant with what the OCT imaging found. However, due to the limitations of CT scan resolution, the TM is not able to be clearly visualized, so the distance between the mass and TM cannot be accurately measured on the CT scan. OCT has a better resolution, and so the distance of the tumor to the TM could be measured, using the index of refraction in air of 1.0 as this distance in the middle ear space is air, and was found to range from 0 to 0.2 mm for the largest distance from the tumor beneath the TM (indicated by the yellow line) in the XZ cross-sectional image (Figure 3E). The fact that there is a space present has good implications for surgical resection. The gap between the TM and the tumor makes it more likely that the TM can be preserved during tumor resection and need not be reconstructed, thus improving the patient’s hearing after surgery.

#### 3.1.3. Cholesteatoma

**Figure 4:**
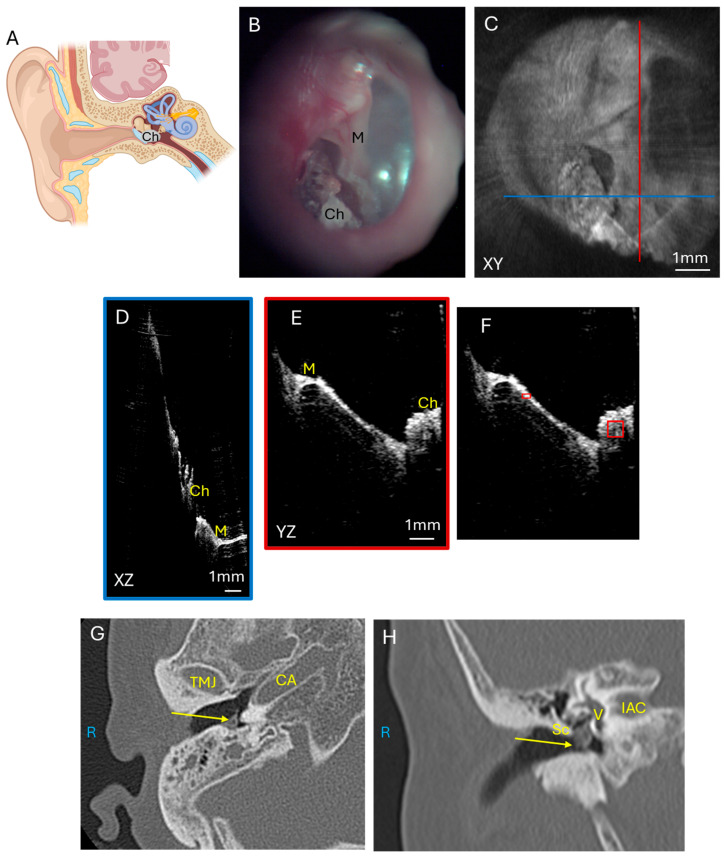
Cholesteatoma. (**A**) Illustration showing the growth of cholesteatoma which usually originates from the superior portion of the tympanic membrane and erodes the ossicular chain and tegmen bone to cause intracranial complications. Endoscopic view of the right tympanic membrane (**B**) of a patient with a mesotympanic cholesteatoma. OCT enface XY view (**C**) of the tympanic membrane, with the blue line representing the plane leading to the middle ear for the cross-sectional XZ (**D**) view (blue box) and the red line representing the plane leading to the middle ear for the cross-sectional YZ (**E**) view (red box) showing the malleus and cholesteatoma. Quantification of cholesteatoma pixel intensity (**F**) (larger red box) in comparison to tympanic membrane pixel intensity (smaller red box). Axial CT (**G**) and coronal CT (**H**) show cholesteatoma (arrow) in the mesotympanic space. R, right side; M, malleus; Sc, scutum; Ch, cholesteatoma; TMJ, temporomandibular joint; CA, carotid artery; V, vestibule; IAC, internal auditory canal.

As glomus tumors are highly vascular, we compared this tumor with cholesteatomas, which are a collection of keratinaceous squamous debris in the middle ear that is avascular. Cholesteatomas (Figure 4A) originate from the tympanic membrane growing into the middle ear and are a collection of epithelial cells that can erode through bone to cause complications such as hearing loss, facial paralysis, and intracranial infections. Figure 4 shows a mesotympanic cholesteatoma through an endoscopic image (Figure 4B) and the corresponding OCT enface XY (Figure 4C) view at the level of the TM. The blue line corresponds with the plane going into the middle ear for the XZ (Figure 4D) cross-sectional view, and the red line corresponds with the plane going into the middle ear for the YZ (Figure 4E) cross-sectional view. The cholesteatoma mass is well visualized in relation to the malleus umbo in the XZ (Figure 4D) and YZ (Figure 4E) views. However, the soft tissue mass attenuates the laser signal and deeper structures such as the incudostapedial joint and cochlear promontory are not well visualized beyond the cholesteatoma. Quantification of the pixel intensity in the cholesteatoma region of interest as normalized to tympanic membrane pixel intensity is shown (Figure 4F). Axial CT (Figure 4G) and coronal CT (Figure 4H) demonstrate the soft tissue mass of the cholesteatoma in the mesotympanum. As many cholesteatomas originate with retraction pockets in the epitympanum, or the superior aspect of the middle ear, our other three patients with cholesteatoma have attic retraction in the posterior superior portion of the TM. Some of these cholesteatomas were extensive enough to have soft tissue signal behind the tympanic membrane in the mesotympanic space as well. If the cholesteatoma was more confined to the epitympanic space, then the region of interest for pixel intensity quantification was defined around the area of soft tissue signal in the epitympanum.

#### 3.1.4. Facial Nerve Schwannoma

**Figure 5:**
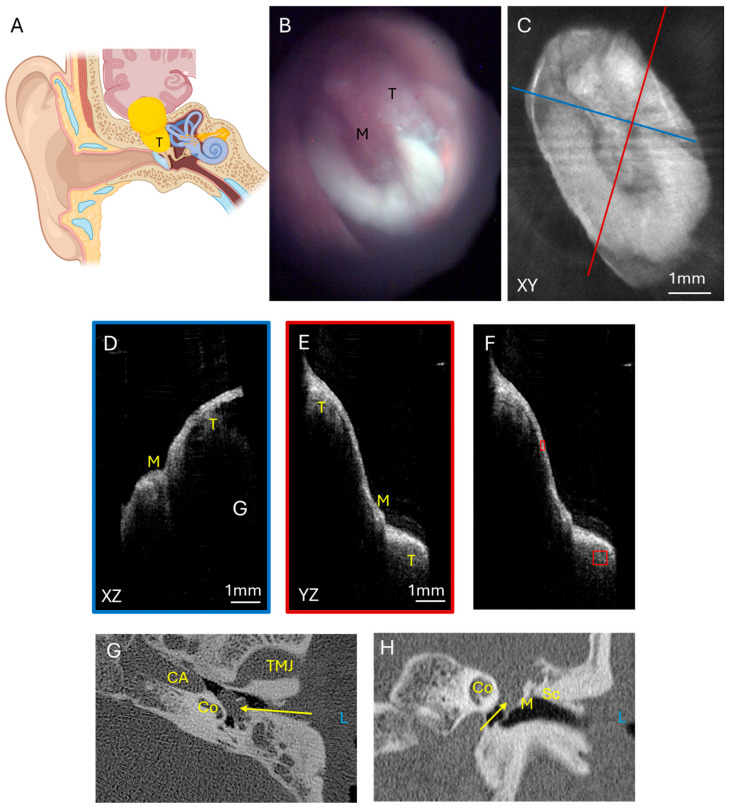
Facial nerve schwannoma. (**A**) Illustration of a facial nerve schwannoma which can arise from multiple areas of the facial nerve course from the brainstem through the middle ear and mastoid bone. These tumors can cause temporal lobe compression and contact the ossicular chain to cause hearing loss and can be visible through the tympanic membrane. Endoscopic view of the left tympanic membrane (**B**) of a patient with a facial nerve schwannoma with a large component of the tumor in the epitympanum and mesotympanum. OCT enface XY view (**C**) of the tympanic membrane, with the blue line representing the plane leading to the middle ear for the cross-sectional XZ (**D**) view (blue box) and the red line representing the plane leading to the middle ear for the cross-sectional YZ (**E**) view (red box) showing the malleus and tumor. Quantification of tumor pixel intensity (**F**) (larger red box) in comparison to tympanic membrane pixel intensity (smaller red box). Axial CT (**G**) showing the tumor (arrow) in the mesotympanic space, and coronal CT (**H**) showing the tumor (arrow) in the epitympanic and mesotympanic space. L, left side; M, malleus; Sc, Scutum; T, tumor; TMJ, temporomandibular joint; CA, carotid artery; Co, cochlea.

A comparison for a tumor with intermediate vascularity is a facial nerve schwannoma with a large middle ear component. Schwannomas are another type of benign tumor that originates in the nerve sheath and can have vascularity but not to the extent of a glomus tumor. The facial nerve courses through the middle ear and mastoid bone to innervate the muscles of facial expression, and facial nerve schwannomas (Figure 5A) can also present with a middle ear mass, temporal lobe compression, and hearing loss. These benign tumors can have mild vascularity but not to the extent of glomus tumors. The left tympanic membrane endoscopic view (Figure 5B) has some myringosclerosis, or scarring, represented by the white area on the TM, which could be due to childhood ear infections, and is unlikely to be related to the facial nerve schwannoma. This TM and ear exam could be considered within the range of normal by a general clinician, though this middle ear contains a facial nerve schwannoma with intracranial extension. The corresponding OCT enface XY view (Figure 5C) at the level of the TM is shown. The blue line corresponds with the plane going into the middle ear for the XZ (Figure 5D) cross-sectional view, and the red line corresponds with the plane going into the middle ear for the YZ (Figure 5E) cross-sectional view. The XZ and YZ views show the relationship of the malleus and extensive soft tissue signal intensity beneath the tympanic membrane. Similar analysis of pixel intensity of the soft tissue tumor as normalized to the tympanic membrane pixel intensity was performed (Figure 5F) for the OCT images of the middle ear component of the facial nerve schwannoma. The axial CT (Figure 5G) and coronal CT (Figure 5H) images show the soft tissue mass nearly filling the middle ear space and extending superiorly to the middle fossa. Without these CT images a priori, a standard otomicroscopy exam, much less an exam with a handheld otoscope, likely would not have identified that a mass was present without additional clinical information. Additionally, this example demonstrates that OCT can penetrate through myringosclerosis to detect the presence of a mass in the middle ear. Therefore, this example demonstrates that OCT can serve as a useful adjunct to otomicroscopy to determine the presence of a middle ear mass.

### 3.2. Volumetric OCT Quantitative Results

**Figure 6:**
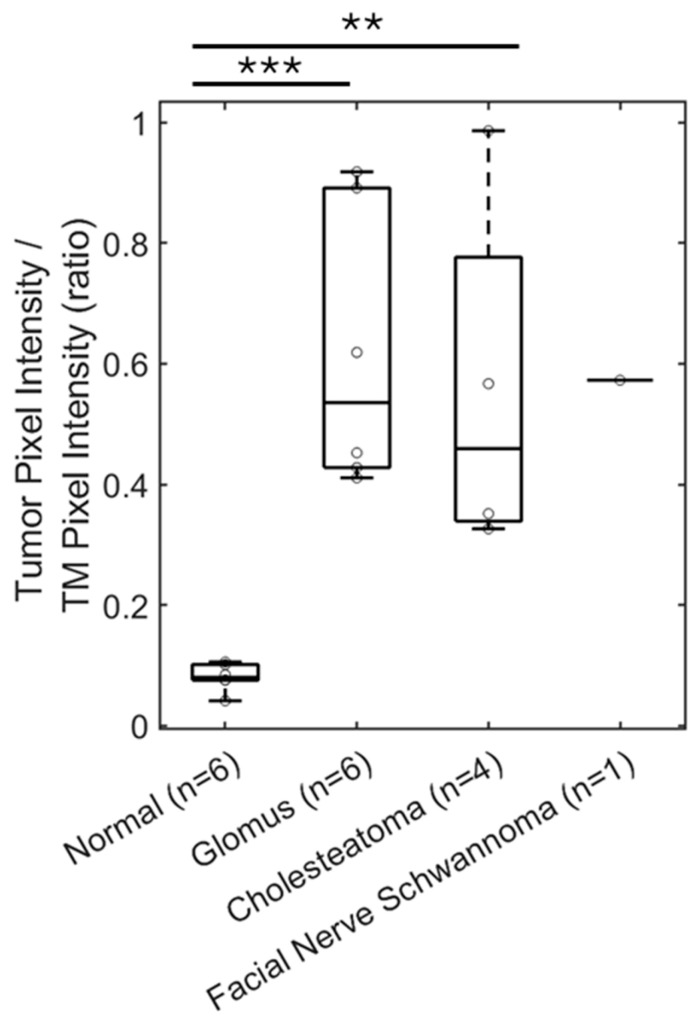
OCT can detect soft tissue masses in the middle ear. The ratio of pixel intensity in the mesotympanum in reference to TM pixel intensity is a measure of tissue density in the middle ear space. This ratio is significantly increased by five-fold for patients with a glomus tumor (*n* = 6), compared to patients with a normal ear exam (*n* = 6). Similar findings are seen with measuring pixel intensity in the epitympanum or mesotympanum for patients with cholesteatoma (*n* = 4) and facial nerve schwannoma with a middle ear component (*n* = 1). TM, tympanic membrane. ** *p* < 0.01, *** *p* < 0.001.

Statistical analysis of the mean pixel intensity in the region of interest with the middle ear masses, normalized to the mean pixel intensity of the tympanic membrane is shown in Figure 6. Glomus tumors, cholesteatomas, and the facial nerve schwannoma all had similar values, demonstrating that OCT can detect the density of the soft tissue mass, compared to the normal middle ear filled with air. The mean pixel intensity ratio was 0.620 ± 0.095 (mean ± standard error) for glomus tumors (*n* = 6), 0.558 ± 0.153 for cholesteatomas (*n* = 4), and 0.573 for facial nerve schwannoma (*n* = 1), which were not significantly different (*p* = 0.72, *t*-test between glomus tumor and cholesteatoma). However, there was a statistically significant difference between the mean pixel intensity ratio of 0.082 ± 0.008 for the group of normal ears (*n* = 6) and the group of glomus tumors (*p* < 0.001, *t*-test) or cholesteatomas (*p* < 0.01, *t*-test). Therefore, volumetric OCT alone can demonstrate the presence of a soft tissue mass in the middle ear but not necessarily the type of middle ear mass.

### 3.3. OCT Angiography

**Figure 7:**
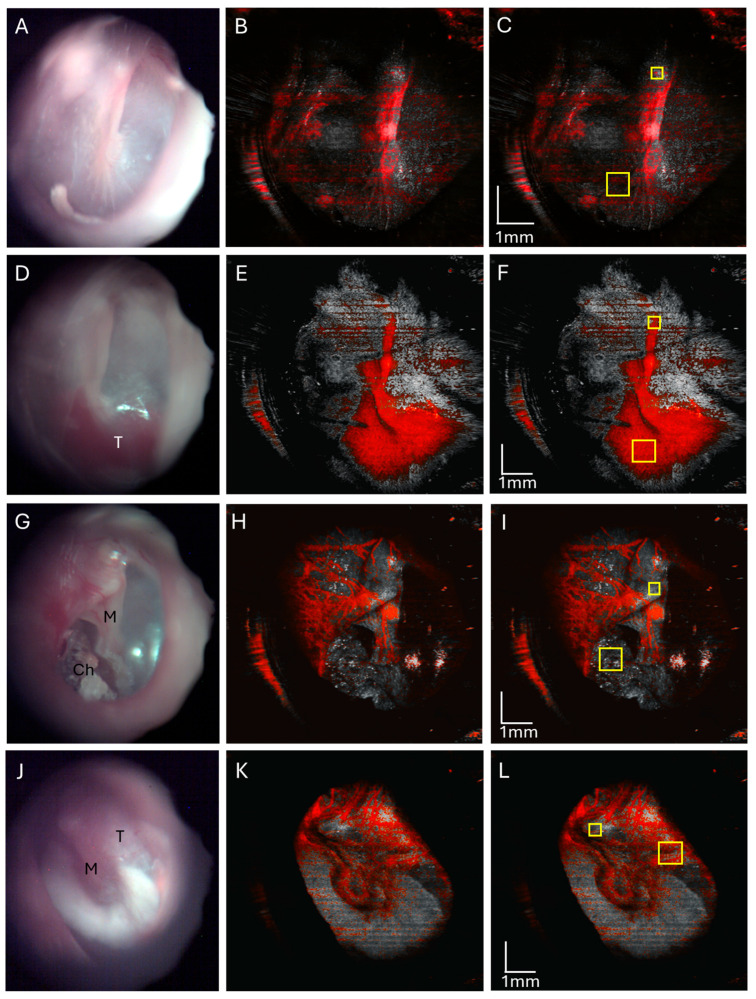
OCT angiography of a normal ear (**A**–**C**), ear with glomus tumor (**D**–**F**), ear with cholesteatoma (**G**–**I**), and facial nerve schwannoma (**J**–**L**). Normal right ear endoscopic view (**A**) with corresponding OCT angiography (**B**) showing normal tympanic vasculature over the area of the malleus, and quantification of the angiography signal (**C**) in the mesotympanum (large yellow box) normalized to the malleus lateral process (small yellow box). Endoscopic view of the right tympanic membrane of a glomus jugulare tumor (**D**) and an OCT angiography overlay (**E**) with a greatly increased angiographic signal in the tumor area, and quantification of the angiography signal (**F**) in the mesotympanic region of tumor (large yellow box) normalized to the malleus lateral process (small yellow box). Endoscopic view of right ear mesotympanic cholesteatoma (**G**) with an OCT angiography signal overlay (**H**) of this mesotympanic cholesteatoma showing the absence of the angiographic signal in the cholesteatoma, and quantification of the angiography signal (**I**) in mesotympanic cholesteatoma (large yellow box) normalized to the malleus lateral process (small yellow box). Endoscopic view of left ear facial nerve schwannoma (**J**) with an OCT angiography overlay (**H**) showing vascularity but less than that of glomus tumors, and quantification of the angiography signal intensity (**I**) (large yellow box) normalized to the malleus lateral process (small yellow box). M, malleus; T, tumor; Ch, cholesteatoma.

However, we found that OCT angiography can differentiate between these middle ear masses. A normal ear endoscopic view (Figure 7A) with an OCT angiography signal overlay in red (Figure 7B) shows the typical tympanic membrane vasculature with the angiography signal in the vascular strip. Note that this example of a normal ear (Figure 7A) does not quite have as translucent a TM as our first example of a normal ear (Figure 1B), which limits evaluation of the middle ear in current standard clinical examination with otoscopy. A glomus jugulare tumor endoscopic view has a prominent red hue behind the tympanic membrane (Figure 7D) with an OCT angiography signal overlay (Figure 7E) showing a high density of vasculature in the region of the tumor in the mesotympanum. A mesotympanic cholesteatoma endoscopic view (Figure 7G) with an OCT angiography signal overlay (Figure 7H) shows an absence of vasculature in the region of cholesteatoma as expected of the avascular keratin debris. The facial nerve schwannoma endoscopic view (Figure 7J) with an OCT angiography signal overlay (Figure 7K) shows some vasculature in the region of the tumor in the epitympanic region but not the high density seen for glomus tumors.

**Figure 8:**
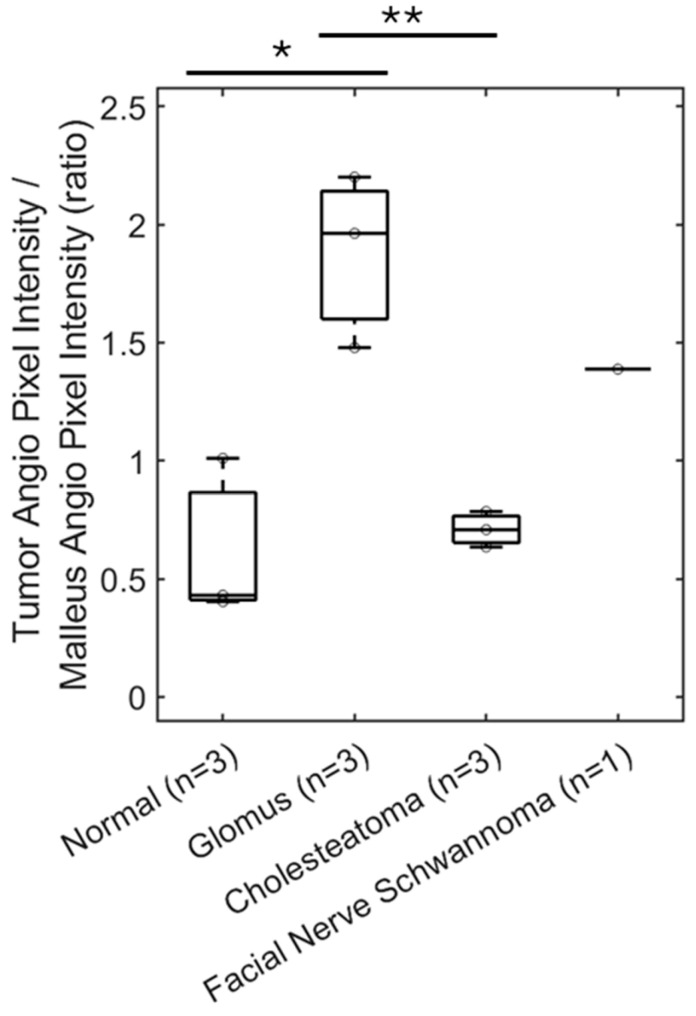
OCT can demonstrate vascularity. Quantification of OCT angiography pixel intensity in the region of the glomus tumor (*n* = 3) shows a significant—almost three-fold—increase compared with cholesteatoma (*n* = 3) or a similar area of a normal ear (*n* = 3). Facial nerve schwannoma has some vascularity but not to the extent of glomus tumors. * *p* < 0.05, ** *p* < 0.01.

The OCT angiography signal, as quantified by mean pixel intensity in the soft tissue mass area of interest, was normalized with the mean pixel intensity over the lateral process of the malleus as this area often contains an angiography signal from vessels of the vascular strip and is a readily identifiable landmark. The area of the quantified angiography signal and area of the malleus lateral process are delineated for a representative normal ear (Figure 7C), glomus tumor (Figure 7F), and cholesteatoma (Figure 7I) for a total sample size of three ears, as well as the facial nerve schwannoma (Figure 7L). The OCT angiography signal for glomus tumors (mean 1.881, standard error 0.212, *n* = 3) was significantly higher and different compared to cholesteatomas (mean 0.709, standard error 0.043, *n* = 3, *p* < 0.01, *t*-test) and normal ears (mean 0.615, standard error 0.198, *n* = 3, *p* < 0.05, *t*-test) as shown in Figure 8. There was no significant difference between the OCT angiography signal for cholesteatomas and normal ears (*p* = 0.67, *t*-test). Interestingly, the OCT angiography signal was intermediate at 1.388 for the facial nerve schwannoma with a middle ear component, suggesting some tumor vascularity but not nearly the vascularity expected in glomus tumors. Therefore, OCT angiography can distinguish the hypervascularity present in glomus tumors from the avascular cholesteatomas and the typical vascularity in normal ears.

## 4. Discussion

Here, we show that a handheld OCT device, which resembles an otoscope used to visualize the tympanic membrane, can rapidly and noninvasively image the tympanic membrane and middle ear through the ear canal in a clinic setting. We have previously reported on the design of this device and the ability of our device to detect chronic otitis media with effusion, tympanic membrane perforations and retractions, and myringitis [6]. In our examples in this report, we demonstrate that OCT is able to image through the tympanic membrane and identify pathology in the middle ear even when the TM is opaque and limits the clinical evaluation of the middle ear. Thus, OCT imaging can determine whether a middle ear mass is present. However, the image pixel intensity alone does not distinguish between different types of masses. By adding OCT angiography, though, the quantification of the vascularity within the mass provides critical distinguishing information. This allows the clinician to differentiate a vascular tumor from a nonvascular mass, providing additional details regarding the pathology. Therefore, this technology has the potential to assist clinicians in identifying and supporting differential diagnoses of middle ear pathology, particularly on the day of initial consultation.

These results demonstrate the feasibility of using an OCT device in the outpatient clinic setting to characterize middle ear pathology such as tumors and masses [10,11,12,13,14]. Currently, most OCT devices used in the clinic for the ear on human patients have been limited to (1) detecting middle ear effusions or (2) imaging the tympanic membrane [7,15,16,17,18,19,20,21]. A commercially developed OCT device with applications limited to determining the presence of a middle ear effusion has proven to be more sensitive than traditional otomicroscopy in establishing whether fluid is present behind the TM in otitis media or chronic otitis media with effusion [10,11,12,13,14,17,22]. The tympanic membrane is the most readily accessible structure for OCT imaging through the ear canal, and a number of groups have built devices and reported on imaging the healthy TM [16,17,18,19,20,21]. Though the TM does attenuate some of the OCT laser signal intensity, high resolution of the middle ear ossicles and ossicular reconstruction prosthesis is possible in human patients with devices built in our lab and other groups [2,7,15,23,24]. However, the current literature regarding imaging pathology in the middle ear in human patients is limited to intraoperative imaging of cholesteatomas after the surgical exposure has been completed [25]. Here, we show that middle ear pathology can be identified and can provide increased resolution of the mass in relation to the tympanic membrane compared to CT scans, with opportunities for quantitative measurements over time to track, for example, the distance of the mass to the TM. The size of a middle ear mass itself can also be quantitated accurately in the X and Y axes but could have uncertainty in measurements in the Z, or depth, dimension due to the refractive index of tissue. The refractive index could vary between 1.33 and 1.45 depending on soft tissue and tumor type [8,26,27], and assuming a refractive index in tissue of 1.4 introduces an uncertainty of approximately 3% in the depth dimension. However, this does not invalidate the ability to compare quantitative measurements for a mass in a patient over time.

We also describe a novel use of OCT angiography for ear imaging in human patients in clinic to distinguish between middle ear masses. For the ear, OCT angiography has only been described for imaging cochlear blood flow in rodent animal models after surgical exposure of the cochlea [28,29,30,31]. However, OCT angiography has been extensively used and established in ophthalmology for retinal imaging to diagnose and monitor diseases involving the microvasculature such as macular degeneration and diabetic retinopathy [9,32]. It has also been used in dermatology in a single study to evaluate three subungual glomus tumors [5], which has a pathology type similar to the glomus tumors in the ear we included in our study. Glomus tumors, including both glomus jugulare and glomus tympanicum, as well as facial nerve schwannomas, are rare entities. Therefore, our sample is small but can be considered adequate given the rarity of these tumors. Furthermore, the consistency of the soft tissue signal on OCT volume images in middle ear pathology, including ears with glomus tumors, cholesteatomas, and facial nerve schwannomas, as compared to normal ears is significantly different and obscures the incudostapedial joint and cochlear promontory with extensive disease. Further improvements on this device are ongoing, including depth of imaging, image acquisition algorithms, motion correction, and more rapid image processing techniques.

The small footprint of the HHOCT device on a mobile endoscopy cart allows it to be housed in the clinic space and be readily available to use, unlike a CT or MRI scanner, which typically requires a large room and is often in a separate building in an academic medical center or hospital setting. The speed of image collection is such that OCT imaging can be readily incorporated into a clinic visit, adding minimal time as scans can be completed within seconds. The documentation and quantification of these OCT three-dimensional volumetric and angiography images could be collected and conducted for every patient in otology clinics in a future study to build a database of normal ears and ears with various pathologies. This database could be used in artificial intelligence and machine learning algorithms to determine if new patients have a normal or abnormal ear exam, and potential differential diagnoses for ear pathology. Then, our HHOCT technology can be broadened for use in primary care and general otolaryngology clinics for patients with ear and hearing-related complaints, to aid these clinicians in a decision of when to order additional cross-sectional imaging such as computed tomography (CT) or magnetic resonance imaging (MRI) and refer patients to further subspecialty care such as at a neurotology clinic. This could help prevent delays in diagnosis and time to care for patients with middle ear pathology, especially pertaining to skull base tumors and middle ear masses, for which delayed diagnosis and treatment could potentially lead to substantial complications.

## 5. Conclusions

OCT is able to image through the TM and detect middle ear masses. OCT angiography correctly assesses the vascularity within these masses. Thus, OCT permits the clinician to have additional point-of-care data that can help make the correct diagnosis.

## Data Availability

Due to the size of the datasets, data supporting the findings of this study are available from the corresponding author upon reasonable request.

## Supplementary Materials

The following supporting information can be downloaded at https://www.mdpi.com/article/10.3390/diagnostics15060732/s1, Table S1: Ears imaged, including pathology and quantification of middle ear mass and vascularity.
