# Supplementary material for: Optical Coherence Tomography Imaging and Angiography of Skull Base Tumors Presenting as a Middle Ear Mass in Clinic
Source: Diagnostics (Basel). 2025 Mar 14;15(6):732. doi: 10.3390/diagnostics15060732 (PMC11941209; doi:10.3390/diagnostics15060732)
Supplement: Supplementary file 1 [file diagnostics-15-00732-s001.zip › diagnostics-3433348-supplementary.pdf]

Table S1. Ears imaged, including pathology and quantification of middle ear mass and vascularity.

Patient #	Pathology	Ear	Race	Treatment	Pixel Intensity (tumor)	Pixel Intensity (TM)	PI(tumor)/PI(TM)	Angio Pixel Intensity	Angio Malleus PI	AngioPI/Malleus PI
1	glomus jugulare	left	African American	surgical resection	82.567	133.429	0.619			
2	glomus jugulare	Left	White	s/p XRT 2018	84.178	94.444	0.891	28376.007	19188.510	1.479
3	glomus tympanicum	right	unknown	observation	57.989	128.219	0.452			
4	glomus jugulare	left	White	surgical resection	76.850	83.700	0.918			
5	glomus jugulare	right	White	observation	108.883	254.500	0.428	20052.555	10217.863	1.962
6	glomus jugulare	left	Asian	s/p XRT	99.576	242.417	0.411	27675.038	12576.992	2.200
7	normal	left	Hispanic	n/a	9.902	93.848	0.106			
8	normal	left	unknown	n/a	18.820	249.033	0.076			
9	normal	right	unknown	n/a	11.179	148.692	0.075			
10	normal	right	Asian	n/a	9.330	186.723	0.050			
11	normal	right	Asian	n/a	10.007	98.857	0.101			
12	normal	left	unknown	n/a	11.390	135.312	0.084			
13	normal	right	Asian	n/a				6671.540	16533.023	0.404
14	normal	right	Asian	n/a				10219.159	10114.814	1.010
15	normal	left	Asian	n/a				11582.578	26897.589	0.431
16	attic cholesteatoma	left	Hispanic	surgical resection	66.162	202.747	0.326			
17	attic cholesteatoma	right	unknown	surgical resection	152.292	154.400	0.986	19945.731	25405.034	0.785
18	mesotympanic cholesteatoma	right	Asian	surgical resection	107.587	189.781	0.567	12531.583	17701.743	0.708
19	attic cholesteatoma	left	Asian	surgical resection	63.036	179.361	0.351	11873.807	18708.450	0.635
20	facial nerve schwannoma	left	Hispanic	surgical resection	80.267	140.150	0.573	28332.522	20415.761	1.388
